# Roles of the co-culture of human umbilical cord Wharton’s jelly-derived mesenchymal stem cells with rat pancreatic cells in the treatment of rats with diabetes mellitus

**DOI:** 10.3892/etm.2014.1985

**Published:** 2014-09-22

**Authors:** GUANGYU WANG, YONG LI, YU WANG, YU DONG, FU-SHENG WANG, YI DING, YUDONG KANG, XUYING XU

**Affiliations:** Ulcerous Vascular Surgical Department, Beijing TCM Hospital Affiliated to Capital Medical University, Beijing 100010, P.R. China

**Keywords:** umbilical cord, Wharton’s jelly, mesenchymal stem cells, transplantation, diabetes mellitus

## Abstract

The aim of the present study was to investigate the roles of the co-culture of human umbilical cord Wharton’s jelly-derived mesenchymal stem cells (hUC-MSCs) with rat pancreatic cells in the treatment of rats with diabetes mellitus. hUC-MSCs were isolated and passaged, followed by Transwell co-culture with rat pancreatic cells. The induced islet-like cell clusters were transplanted into the renal capsule in rats with streptozotocin (STZ)-induced diabetes mellitus. The effects of co-culture on blood glucose levels in rats were observed. The isolated hUC-MSCs expressed the specific surface markers, including cluster of differentiation 44 (CD44) (91.4%), CD29 (91.3%) and CD105 (99.2%). Following co-culture with hUC-MSCs for 7 and 10 days, the rat pancreatic cells were strongly stained by pancreatic and duodenal homeobox-1 and human insulin. The insulin and C-peptide concentrations were increased significantly compared to the pure culture group. One week following the transplantation of induced islet-like cells into the renal capsule, the blood glucose level of rats in the STZ experimental group was significantly lower than that of the STZ control group. There were notable 5-bromo-2′-deoxyuridine-positive nuclei and insulin-positive cytoplasm in the renal capsule following cell transplantation. Therefore, co-culture of hUC-MSCs with rat pancreatic cells can lower the blood glucose levels in rats with diabetes mellitus.

## Introduction

Replacement therapy with stem cells has become a novel method for the treatment of type 1 diabetes mellitus (T1DM). As confirmed by *in vitro* experiments, embryonic stem cells (1,2), induced pluripotent stem cells (skin fibroblasts, pancreatic cells, spermatogonia) (3,4), hepatic stem cells (hepatic oval cells, bile duct cells, white blood cells) (5–7), pancreatic stem cells (pancreatic duct cells, islet cells, exocrine cells) (8,9), and mesenchymal stem cells (MSCs) (including bone marrow, fat, umbilical cord and cord blood) (10,11) can be induced to differentiate into cells with insulin secretion function. However, when applied in clinical practice, the sufficiency of the cell source and a series of problems, including ethics, immunogenicity and subculture, should also be considered. Clearly, the umbilical cord-derived MSCs have irreplaceable advantages, and are attracting increasing clinical attention.

Thus far, selecting appropriate methods to transplant stem cells is one study foci. In animal experiments, the common stem cell transplantation methods include orthotopic transplantation (12), renal subcapsular transplantation (13), subcutaneous transplantation (14), intravenous transplantation (15), transplantation in the portal vein (16) and transplantation in the testes (17). However, these methods will encounter a number of difficulties when applied in clinical practice. Due to the progress of interventional technology, the small arteries, including the dorsal pancreatic artery, can be selected for intervention. If the stem cells are injected though the dorsal pancreatic artery, they may be induced to differentiate into cells with an insulin secretion function under the pancreas microenvironment. This has attracted increasing clinical attention, and has provided a combining site of basic research and clinical application.

In the present study, the human umbilical cord-derived MSCs (hUC-MSCs) were co-cultured with rat pancreatic cells. Their induced differentiation into islet-like cells was observed. These cells were transplanted into diabetic rats with diabetes mellitus, and their effects on blood glucose in rats were investigated. The objective was to provide a novel proposal for applying stem cells to the treatment of T1DM.

## Materials and methods

### Materials

The experiment was performed at the Bethune International Peace Hospital (Shijiazhuang, China) between January 2009 and December 2010. The umbilical cord was obtained from the first delivery healthy parturient, pregnancy at term, and only when the tests for hepatitis B, syphilis or acquired immunodeficiency syndrome were negative. The study was conducted in accordance with the Declaration of Helsinki and with approval from the Ethics Committee of Beijing Traditional Chinese Medicine Hospital Affiliated to Capital Medical University (Beijing, China). Written informed consent was obtained from all the participants.

Pancreatic cells were obtained from 60 male Sprague Dawley (SD) rats, weighing 220 g. Another 30 male rats, aged 8 weeks, weighing 180–220 g, were prepared. All the animals were supplied by Hebei Experimental Animal Center (Shijiazhuang, China; certificate no. 911141).

### Cell culture

Cord blood was washed with stroke-physiological saline solution, the umbilical vein and artery were removed followed by cutting into 1.0 to 2.0-cm segments in the laminar flow chamber. The Wharton’s Jelly was separated, smashed into 1×1×1-mm size and cultured with Dulbecco’s modified Eagle’s medium (DMEM)/F12 medium in an aseptic culture flask at 37°C in a 5% CO_2_ incubator. One week later, the supernatant was removed and the medium was replaced every 3–4 days. When the adhered cells reached 80%–90% confluency, trypsin/EDTA was added to digest and passage the cells. The cells were passaged for 11 generations.

### hUC-MSCs surface markers

The 3–5 generation adhered cells were digested with 2.5 g/l trypsin, and when the cells were nearly round under an inverted microscope, DMEM/F12 medium containing 10% fetal bovine serum (FBS) was added to terminate the digestion, followed by 5 min centrifugation at 840 × g for 5 min and twice washing with phosphate-buffered saline (PBS). PBS-balanced solution (100 μl) was added to prepare the cell suspension. Mouse-anti-human monoclonal and negative-control antibodies were added (all purchased from Sigma-Aldrich, St. Louis, MO, USA): i) Cluster of differentiation 105-phycoerythrin (CD105-PE) and immunoglobulin G (IgG)1k-PE. ii) CD29-fluorescein isothiocyanate (FITC) and IgG2a-FITC. iii) CD44-FITC and IgG1-FITC. iv) CD14-FITC and IgG2ak-FITC. v) CD34-PE and IgG1k-PE. vi) CD45-PE-cyanine 5 (PECY5) and IgG1-PECY5. The cells were incubated for 30 min at room temperature, washed twice with PBS following centrifugation, followed by the addition of FITC cross-linked rat-anti-mouse second antibody (1:400). Subsequent to culture for 45 min, the cells were resuspensed with 3 ml PBS, washed with PBS at 840 × g for 5 min and resuspended in 300 μl PBS. The cell suspension was filtered and analyzed using flow cytometry. All the procedures were repeated three times.

### MTT

The passages of three, five, seven, nine and 11 generations of hUC-MSCs were incubated in 96-well culture plates with a 100 μl density and 1×10^4^ cells per well. Each passage of cells was cultured in six wells, and the culture medium was set for the blank control. All the cells were routinely cultured in the incubator. MTT (10 μl; 5 g/l) was added and the cells were cultured for another 4 h. The supernatant was removed, followed by the addition of 150 μl dimethylsulfoxide, shocking for 10 min and measuring the absorbance value (*A*) at 492 nm. The cell growth curve was drawn with time as the X-axis and *A* value as the Y-axis. *A* value was expressed as the mean ± standard deviation.

### Cells separation

Male SD rats, weighing 220 g, were anesthetized using 10 g/l pentobarbital with 45 mg/kg. At the dorsal position, an arrow-shaped incision was performed at the abdominal section and the abdominal cavity was opened. The common bile duct was ligated at the approaching duodenum site using no. 1 silk suture. A no. 4.5 intravenous needle was inserted into the common bile duct near the hepatic porta and fixed using a silk suture. The rats were sacrificed by opening the hearts and bloodletting. A total of 8 ml pre-cooled collagenase V solution (0.5 g/l) was injected into the pancreas to make it expand. The harvested pancreas was placed in the digestion bottle with 6 ml D-Hank’s solution. The blood vessel, fat and lymph node were removed, and the pancreas was smashed, followed by incubation in a 30°C incubator for 10 min. Following the removal of the digested tissues, the remaining tissues were continuously digested with collagenase V. Digestion of all the digested tissues was terminated by the addition of 10 ml 4°C FBS and 30 ml 4°C D-Hank’s solution. Following filtration, the cell suspensions were centrifuged with a 50 ml centrifuge tube for 3 min at 728 × g, discharging the supernatant and washing with 4°C normal saline. The procedure was repeated. The deposition was resuspended with low glucose (LG)-DMEM, and 2 ml dithizone was added. Finally, the staining of the cells was observed under an inverted microscope (Olympus CX41; Olympus, Tokyo, Japan).

### Differentiation

The 4–10 passage hUC-MSCs were incubated in the Transwell plate with a density of 1×10^6^ and cultured with 3 ml LG-DMEM containing 10% FBS. The medium was replaced by LG-DMEM containing 10% FBS mixed with RPMI 1640 (1:1) at 3 days following culture. The insert, with a pore diameter of 0.4 μm, was inserted into the plates, and islet-like cells were seeded at 1×10^6^ density to co-culture with hUC-MSCs. The culture medium was renewed every 3–4 days. The cell morphological changes were observed under an inverted microscope. Identification of pancreatic and duodenal homeobox-1 (PDX-1) expression was carried out by cell dithizone staining.

The slides of the cells in the co-culture group and pure-culture group were dried for 3, 7, 10 and 14 days, fixed with 40 g/l paraform for 60 min, washed three times with PBS for 5 min, soaked in 3% hydrogen peroxide for 10–15 min, washed three times with PBS at 5 min per time, followed by the addition of 0.1% TritonX-100, cultured at room temperature for 10 min, washed three times with PBS at 5 min per time, blocked with bovine serum albumin for 10–15 min, followed by the addition of rabbit-anti-human PDX-1 polyclonal antibody (1:50), and incubated overnight in a wet box. The following day, the cells were washed with PBS for 5 min three times, colorized with diaminobenzidine for 5 min, washed five times with double-distilled water, followed by re-staining with haematoxylin for 1 min, dehydration, clearing in xylene and embedding in paraffin. hUC-MSCs prior to and at 3, 7, 10 and 14 days after culture were dried and washed twice with PBS. Dithizone was added and observed under an inverted microscope.

### Reverse transcription polymerase chain reaction (RT-PCR)

The expressions of *PDX-1* and human insulin in hUC-MSCs were detected prior to and at 3, 7, 10 and 14 days after culture as follows. The cells were centrifuged, rinsed three times in PBS and counted. The cell RNA was subsequently extracted using TRIzol^®^ reagent (Invitrogen, Carlsbad, CA, USA). Reaction conditions were: 94°C for 30 sec, 94°C for 30 sec, 58°C for 30 sec and 72°C for 30 sec repeated in total for 30 cycles, followed by 72°C for 5 min. The primers were: Human insulin [192 base pairs (bp)] forward, 5′-TTC TTC TAC ACA CCC AAG AC-3′; reverse, 5′-CTA GTT GCA GTA GTT CTC CA-3′; *PDX-1* (305 bp) forward, 5′-ACC CGT ACA GCC TAC ACT CG-3′; and reverse, 5′-TCA TCG CCC TGT TGC TCG-3′.

### Cells secretion

hUC-MSCs were cultured *in vitro* with a density of 1×10^8^/l in LG-DMEM/RPMI1640 (1:1) containing 82.5 mmol/l glucose. The contents of insulin and C-peptide in the co-cultured and single group were detected by a radioimmunological kit (Human C-Peptide and Human Insulin-Specific radioimmunoassay kits, Linco Research, St. Charles, MO, USA) at 0, 3, 7, 10, and 14 days after culture.

### Glucose-stimulated insulin secretion (GSIS) test

Islet-like cells in the co-cultured group were obtained at 6 days after culture, washed twice using RPMI 1640, and serum-free LG-DMEM (containing 55 mmol/l glucose) and high glucose (HG)-DMEM (containing 247.5 mmol/l glucose) were added, respectively, at 2 ml per well, and incubated in a 5% CO_2_ incubator at 37°C for 2 h. Subsequently, 0.5 ml supernatant was harvested from each well, placed into an Eppendorf tube and maintained at −20°C. Pure cultured cells at 6 days served as controls. The secretion of insulin in the two groups was analyzed by the radioimmunological method, and the glucose stimulation index was calculated as: Glucose stimulation index = HG-DMEM insulin content/LG-DMEM insulin content.

### Model preparation

Male SD rats, aged eight weeks, weighing 180–220 g, were prepared for the diabetic models according to routine methods. Secretion of insulin and C-peptide was detected by the radioimmunological method, and hematoxylin and eosin (HE) staining was used to observe morphological changes of the islets. Blood glucose of the rat caudal vein was measured every other day (Roche Accu-Chek^®^ Advantage blood glucose meter system; Roche, Manheim, Germany), and the body weight was measured every week. All the rats were randomly divided into three groups: Normal control group (n=6), no treatment; model group (n=8), prepared for diabetic models; and experimental group (n=16), transplanted islet-like cells following model preparation.

### Labeling rate

Culture medium for the islet-like cells was renewed at 48 h before transplantation, and 5-bromo-2′-deoxyuridine (BrdU) with a final concentration of 10 μmol/l was added. Trypsin/EDTA were added after 48 h following culture to digest the cells, followed by washing twice using PBS. Subsequently, the cells were prepared for the slides, fixed, and incubated overnight in a wet box with added 1:50 mouse-anti-human BrdU monoclonal antibody. The cells were colorized by a chromogenic agent, and the positive cells were stained blue. The coloration duration was controlled under a microscope.

### Transplantation

BrdU-labeled islet-like cells were digested by trypsin/EDTA, centrifuged, washed twice by PBS and prepared for single cell suspension. The cell concentration was adjusted for 5×10^9^/l. The rats were anesthetized by 10 g/l pentobarbital with 45 mg per kilogram, and fixed on the surgical table in the prone position. The surgical area was sterilized using 75% ethanol. A lateropulsion incision was cut along the left costal margin, and the skin was cut to expose the left kidney. The prepared cell suspension (1 ml) was injected into the capsule at the lower pole of the left kidney. The kidney was replaced, and the skin was sutured and sterilized with 75% ethanol. All the rats were maintained under thermal insulation until conscious.

### Observation

All the rats were observed for eight weeks after transplantation. The blood glucose was measured and the animals were sacrificed following observation. The kidney was cut into slices, and stained by human insulin and BrdU to observe the local transplantation outcomes.

### Main outcome measures

i) Surface marker for stem cells. ii) Morphology, dithizone and PDX-1 staining of hUC-MSCS co-cultured with rat islet cells. iii) Changes of *PDX-1* and human insulin mRNA expression during cell differentiation. iv) Secretion of insulin and C-peptide in co-cultured cells. v) Outcomes of the GSIS test.

### Statistics

Data are presented as mean ± standard deviation. One-way analysis of variance (ANOVA) was used to assess significance. The Student-Newman-Keuls method was used to assess the mean values using SPSS 13.0 software (SPSS, Inc., Chicago, IL, USA). P<0.05 was considered to indicate a statistically significant difference.

## Results

### Isolation, culture and passage of umbilical cord Wharton’s jelly-derived MSCs

**Figure 1:**
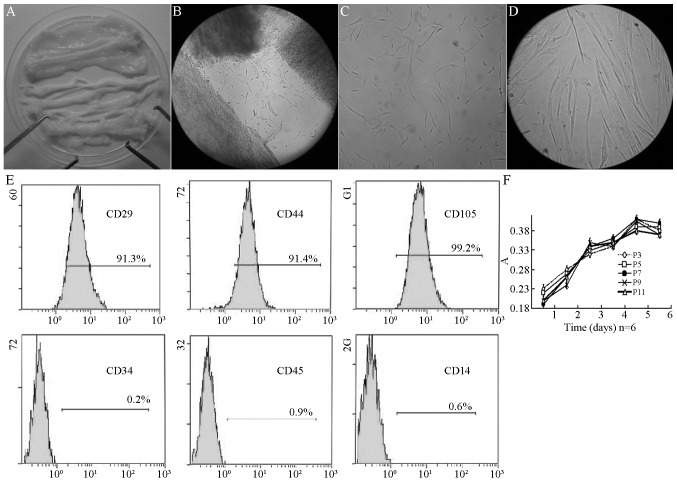
Passage of hUC-MSCs in Wharton’s Jelly. (A) Wharton’s Jelly tissues without umbilical artery or vein. (B) Cells crawled out of cultured Wharton’s Jelly 2 days after culture (magnification, ×100). (C) Fusiform shape cells adhered to the bottle 5 days after culture (magnification, ×100). (D) Typical hUC-MSCs could be observed 7 days after culture (magnification, ×400). (E) The surface marker of human MSCs in Wharton’s Jelly of the human umbilical cord expressed CD44 (91.4%), CD29 (91.3%), CD105 (99.2%), but not CD34 (0.2%), CD45 (0.9%) or CD14 (0.6%). (F) Growth curves of human umbilical cord derived MSCs at 3, 5, 7, 9 and 11 passages. hUC-MSCs, human umbilical cord mesenchymal stem cells; CD, cluster of differentiation.

Following the removal of the vein, the umbilical cord was a type of milky white connective tissue (Fig. 1A). Subsequent to shearing and sieving, the tissue blocks were implanted to the culture medium. Two days later, short spindle cells appeared on the tissue blocks (Fig. 1B). Five days later, 80–90% cells were fused and the adherent cells were obtained, which was the first generation of cells. These long spindle cells had a unique shape of MSCs (Fig. 1C). After 7 days of culture, these cells had a typical appearance of MSCs (Fig. 1D). Subsequent to the first passage, the growth of the cells increased, with one passage for each 3–4 days. There were 11 passages in total. Flow cytometry was conducted. As shown in Fig. 1E, the positive surface marker presented the expression of CD44 (91.4%), CD29 (91.3%) and CD105 (99.2%), respectively, without expression of CD34 (0.2%), CD45 (0.9%) or CD14 (0.6%). The 3rd, 5th, 7th, 9th and 11th generation of cells were obtained and cultured in medium. The cell activity was determined by testing the absorbance. There was no significant difference of absorbance among 6 days of culture (P>0.05) (Fig. 1F).

### Dithizone and PDX-1 immunohistochemical staining in co-culture of hUC-MSCs with rat islet cells

**Figure 2:**
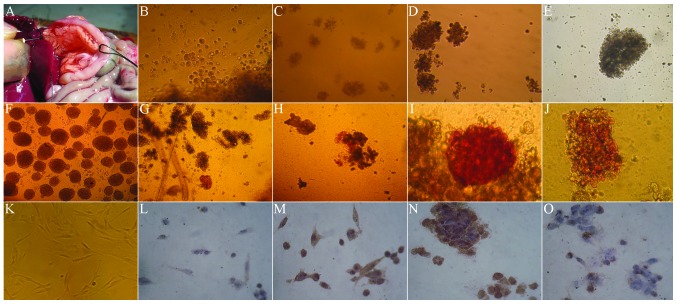
Morphology, dithizone and PDX-1 staining of hUC-MSCs in Wharton’s Jelly co-cultured with rat’s islet cells (magnification, ×100). (A) Extraction of rat islet cells. (B) In the first few days of co-culture with the rat islet cells, hUC-MSCs rapidly became spherical. (C) In the 7 days of co-culture, the spherical cells gradually accumulated and became a large cell mass. (D) In the 10 days of co-culture, the spherical cells gradually accumulated and became a large cell mass. (E) After 14 days, the cells aggregated and became a larger cell mass. (F) The cytoplasm was brownish-red under dithizone staining, indicating strong positive. (G) At day 3 of co-culture with the rat islet cells, the dithizone staining of hUC-MSCs was negative. (H) At day 7 of co-culture with the rat islet cells, the dithizone staining presented strong brownish red. (I) At day 10 of co-culture with the rat islet cells, the dithizone staining presented strong brownish red. (J) At day 14 of co-culture with the rat islet cells, the stained cells were comparatively rare in dithizone staining. (K) Prior to culture, the hUC-MSCs were long fiber-shaped or fusiform-shaped. (L) At day 3 of co-culture with the rat islet cells, the PDX-1 staining of hUC-MSCs was negative. (M) At day 7 of co-culture with the rat islet cells, the PDX-1 staining presented strong brownish. (N) At day 10 of co-culture with the rat islet cells, the PDX-1 staining presented strong brownish. (O) At day 14 of co-culture with the rat islet cells, the stained cells were comparatively rare in PDX-1 staining. PDX-1, pancreatic and duodenal homeobox-1; hUC-MSCs, humal umbilical cord mesenchymal cells.

The digestive enzyme was inversely injected into the common bile duct by intubation (Fig. 2A), for obtaining more rat islet cells. The cytoplasm was brownish red under dithizone staining, indicating strong positive staining (Fig. 2F). Fig. 2K showed that before culture, the hUC-MSCs were long fiber-shaped or fusiform-shaped. In the first few days of co-culture with the rat islet cells, hUC-MSCs rapidly became spherical (Fig. 2B). The dithizone (Fig. 2G) and PDX-1 staining (Fig. 2L) were negative, and there was almost no brown staining in the cytoplasm. In the 7–10 days of co-culture, the spherical cells gradually accumulated, and became a large cell mass (Fig. 2C and D). The dithizone staining presented as a strong brownish-red (Fig. 2H and I), with strong brown in cytoplasm by PDX-1 staining (Fig. 2M and N). After 14 days, the cells aggregated and became a larger cell mass (Fig. 2E), the stained cells were comparatively rare in dithizone staining (Fig. 2J) and PDX-1 staining (Fig. 2O).

### Changes of PDX-1 and human insulin mRNA expressions during induced differentiation in co-culture

**Figure 3:**
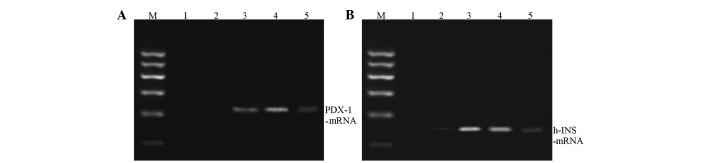
*PDX-1* and human insulin mRNA expressions during induced differentiation in co-culture. (A) *PDX-1*; (B) Human insulin. *PDX-1*, pancreatic and duodenal homeobox-1; M, marker lane.

During co-culture, the *PDX-1* and human insulin mRNA expressions in hUC-MSCs at various time points were determined. The results showed that the *PDX-1* and human insulin mRNA expression was essentially the same at each time point. The expression was lower in the first 3 days, and significantly increased at 7–10 days. At day 14, the expression levels were significantly reduced (Fig. 3).

### Change of insulin and C-peptide level in culture medium during culture

During co-culture, the insulin and C-peptide levels of culture supernatant were detected. As shown in Table I, for MSCs with no co-culture (only in culture medium; pure culture group), the insulin and C-peptide levels did not clearly change during culture (P>0.05). During the co-culture (co-culture group), the insulin concentration in the culture supernatant increased from the initial 2.0254 μIU/ml to 141.1400 μIU/ml at day 7. At day 10 it was 105.7600 μIU/ml and decreased to 57.4533 μIU/ml at day 14, with significant differences among the various time points (P<0.01). There were similar results of C-peptide level. At day 3 of culture, the C-peptide concentration increased by ~20 times, and continued to rise at the first 7–10 day, then decreased gradually. At day 14, the C-peptide concentration remained high, with significant differences among time points (P<0.01).

### Results of the glucose-stimulated insulin release experiment

**Figure 4:**
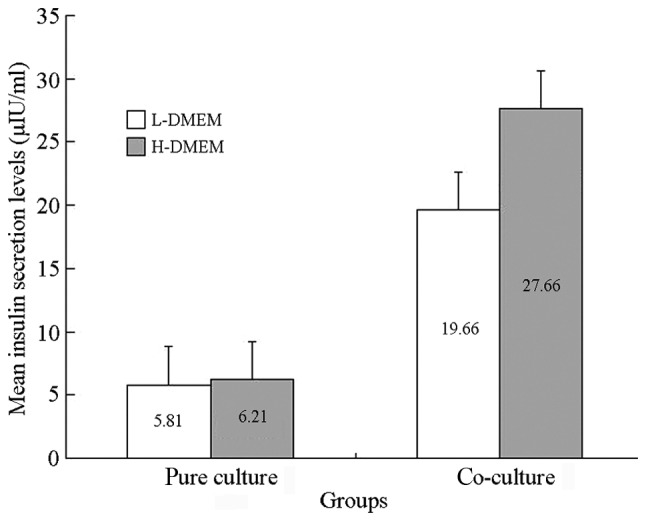
Results of the glucose-stimulated test. L-DMEM, low glucose Dulbecco’s modified Eagle’s medium; H-DMEM, high glucose DMEM.

In the co-culture group, the insulin secretion in LG-DMEM and HG-DMEM was 19.6025±5.9516 μIU/ml and 27.6617±7.1483 μIU/ml, respectively. The secretion significantly increased with an increase of glucose concentration (P<0.01). In the pure culture group, the amount of insulin secretion for the simple culture cells in LG-DMEM and HG-DMEM was 5.8000±1.006 μIU/ml and 6.2100±1.1746 μIU/ml, respectively, with no significant difference between them (P>0.05). This indicated that at day 7 of co-culture, the islet-like cells reacted to the stimulation of glucose, and the glucose stimulation index was 1.4372±0.1390 μIU/ml (Fig. 4).

### Results of BrdU and HE staining

**Figure 5:**
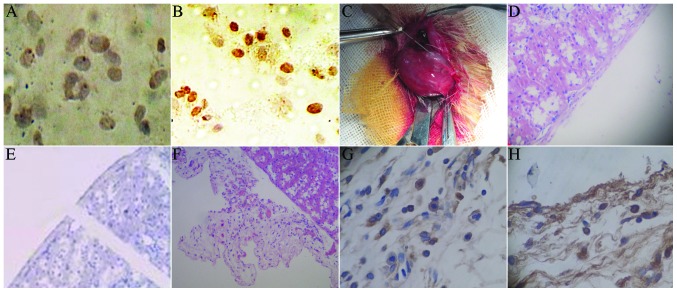
5-bromo-2′-deoxyuridine (BrdU) staining of islet-like cells (magnification, ×250). (A) For cells without BrdU marking, there was no brown staining in the cytoplasm. (B) Light brown staining for BrdU-marked cells. (C) The induced islet-like cell clusters were transplanted into the renal capsule in rats. (D) The HE staining of normal rat kidney showed integral renal capsule edge, with clear morphology and cell structure. (E) The immunohistochemistry of normal rat kidney showed that the insulin and BrdU staining of the renal capsule were negative. (F) Following transplantation with induced islet-like cells, the HE staining of the renal cells showed that there were a large number of surviving cells between the renal capsule and renal cortex, with fracture of the renal capsule. (G) The kidney immunohistochemical staining showed insulin-positive cells between the renal capsule and renal cortex. (H) The immunohistochemical staining indicated BrdU-positive cells between the renal capsule and renal cortex, with brown nuclei. BrdU, bromodeoxyuridine; HE, hematoxylin and eosin

Under the 250× microscope, 10 visual fields were randomly selected to count the number of positive and non-positive cells. The positive rate of BrdU was calculated as: Number of positive cells/(number of positive cells + non-positive cells). The mark rate of BrdU was 52%. For the cells without BrdU marking, there was no brown staining in the cytoplasm (Fig. 5A), with light brown staining for BrdU-marked cells (Fig. 5B). Under sterile conditions, the micro injector was used for local injection to the renal capsule, followed by induced transplantation. The induced islet-like cell clusters were transplanted into the renal capsule in rats (Fig. 5C). The HE staining of normal rat kidney showed an integral renal capsule edge, with clear morphology and cell structure (Fig. 5D). The immunohistochemistry of the normal rat kidney showed that the insulin and BrdU staining of the renal capsule were negative (Fig. 5E). Following transplantation with induced islet-like cells, the HE staining of the renal cells showed that there were a large number of survived cells between renal capsule and the renal cortex, with fracture of renal capsule (Fig. 5F). The kidney immunohistochemical staining showed insulin-positive cells between the renal capsule and renal cortex (Fig. 5G). The immunohistochemical staining indicated BrdU-positive cells between the renal capsule and renal cortex, with brown nuclei (Fig. 5H).

### Blood glucose and body weight change, glucose-stimulated test results and survival curve

**Figure 6:**
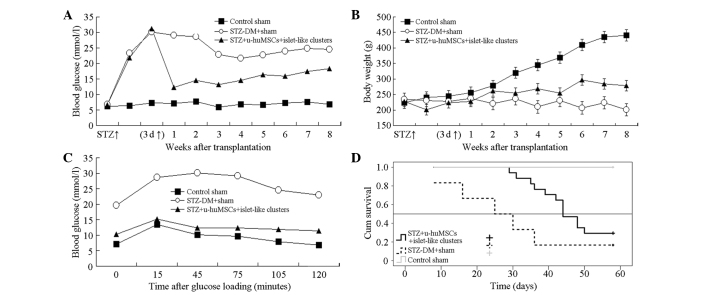
Blood glucose and body weight change, glucose-stimulated test results and survival curve. (A) Blood glucose; (B) Body weight; (C) Glucose-stimulated test; (D) Kaplan-Meier survival curve.

The Roche blood glucose meter detection results (Fig. 6A) showed that in the first week, the blood glucose level in the STZ-experimental group decreased, with significant difference compared to the normal and STZ-control groups (P<0.01). Fig. 6B showed that following treatment the body weight in the normal group significantly increased, while in the STZ-normal group the weight decreased gradually. In the STZ-experimental group, the body weight gradually increased with a significant difference compared to the normal and STZ control groups in the 4, 6, 7 and 8 weeks after transplantation (P<0.01). Kaplan-Meier survival curve of each group following cell transplantation is shown in Fig. 6C. The survival time of the diabetic rats in the STZ-experimental and STZ-control groups was 46.56±2.29 and 28.83±6.49 days, respectively, with a significant difference between them (P=0.026). Fig. 6D shows that the survival rate in the STZ-experimental group was higher compared to the STZ-control group.

## Discussion

Wharton’s Jelly is a type of mucin-like connective tissue in the umbilical cord that contains two umbilical arteries and an umbilical vein. During cell culture, there are long fiber-like or fusiform-shaped adherent cells, which express CD44, CD29 and CD105, but not CD34, CD45 or CD14. From the morphology and cell surface markers, these cells have typical characteristics of MSCs. Therefore, these cells can be regarded as hUC-MSCs.

There are a number of approaches for the differentiation of MSCs into insulin secretion cells, which include *in vitro* induction (18), transgene induction (19) and micro-environmental methods such as low-sugar environment culture (20) and pancreatic extract co-culture (21). Transwell co-culture plate has 0.4 μm microporous semi-permeable membrane chamber. The selective membrane only allows the passage of small molecules from the medium, but not insulin, C-peptide or other large molecules. The isolated rat pancreatic cells were co-cultured with hUC-MSCs, so that the cell cytokine in the islet microenvironment can act with hUC-MSCs through the semi-permeable membrane. This can simulate the microenvironment in the human pancreas more efficiently.

In the present study, the morphological observation, immunocytochemistry, RT-PCR and radioimmunoassay were conducted to observe the potential and functional change trends of hUC-MSCs differentiating into islet-like cells with different *in vitro* induction terms. In the first few days of induction in the islet microenvironment, the shape of hUC-MSCs changed from long fusiform into round shapes. These cells aggregated into clusters, with a semi-suspended state growth, which is similar with *in vitro* cultured islet.

Under physiological conditions, PDX-1 is a critical transcription factor for the early growth of pancreatic tissue. PDX-1 can promote the directional differentiation of pancreatic precursor cells to pancreatic tissue, and promote the cell to secret insulin and C-peptide. With the growth and maturation of the pancreas, the expression of PDX-1 gradually decreases. The basic principle of hUC-MSCS differentiating into islet-like stem cells is that it initiates the expression of PDX-1. In the present study, after 7 and 10 days of induction, the PDX-1 expression in the islet-like cells in the co-culture group was strong positive, and in the pure culture group it was negative. This indicates that in the short induction term (one week), there is PDX-1 expression in cells. In the co-culture system under the microenvironment provided by the rat islet cells, the hUC-MSCs can differentiate to early islet cells. In the co-culture group, the radioimmunoassay detected high-level insulin and C-peptide in the culture supernatant in the day 7 and 10 cells, indicating that the induced cells have the ability to synthesize and secrete insulin, and have reactivity to glucose stimulation with an insulin stimulation index of 1.4372±0.1390. In the pure culture group, only a small amount of insulin was detected in the supernatant. With the prolonged *in vitro* culture time, the secretion amount of insulin had no evident change. This may be caused by the effect of low-concentration glucose in medium on the differentiation of hUC-MSCs. After 10 days of *in vitro* induced culture, the insulin and C-peptide secretion function of hUC-MSCs gradually decreased. At day 14, there was only an extremely small amount of insulin secretion and PDX-1 expression. This is consistent with the RT-PCR results.

The present study shows that in the microenvironment of the co-culture with rat pancreatic cells, the hUC-MSCs can survive, proliferate and be induced to differentiate to islet-like cells. At week 8 after transplantation to the kidney capsule, the cells remained alive. The dithizone staining showed that the cells produced insulin and decreased the blood glucose in diabetic rats. In addition, there was no clear immunological rejection associated with transplantation. The induction does not bring other chemicals and genes. Simultaneously, the hUC-MSCs have superiority in number compared to other stem cells, which fulfills the clinical requirement. The study has provided the experimental basis for the further application of dorsal pancreatic artery injection of hUC-MSCs to the treatment of T1DM.

## Figures and Tables

**Table I:** Comparison of insulin secretion levels between the two groups at different times following induction by radioimmunoassay (n=6).

	Pure cultured group		Co-cultured group	
		
Time, days	Insulin, mU/l	C-peptide, nmol/l	Insulin, mU/l	C-peptide, nmol/l
0	2.03±0.75	0.02±0.01	2.02±0.56	0.02±0.01
3	2.03±0.80	0.01±0.01	65.46±17.30	0.28±0.09
7	2.17±0.76	0.01±0.01	141.14±20.14	0.60±0.15
10	1.98±0.74	0.01±0.01	105.76±18.68	0.44±0.08
14	2.17±0.46	0.02±0.00	57.45±17.06	0.20±0.08
*F*			26.775	17.424
P-value	>0.05	>0.05	<0.01	<0.01

Data are presented as the mean ± standard deviation.

## References

[1] Kroon E, Martinson LA, Kadoya K (2008). Pancreatic endoderm derived from human embryonic stem cells generates glucose-responsive insulin-secreting cells in vivo. Nat Biotechnol.

[2] Chen S, Borowiak M, Fox JL (2009). A small molecule that directs differentiation of human ESCs into the pancreatic lineage. Nat Chem Biol.

[3] Alipio Z, Liao W, Roemer EJ (2010). Reversal of hyperglycemia in diabetic mouse models using induced-pluripotent stem (iPS)-derived pancreatic beta-like cells. Proc Natl Acad Sci USA.

[4] Bar-Nur O, Russ HA, Efrat S, Benvenisty N (2011). Epigenetic memory and preferential lineage-specific differentiation in induced pluripotent stem cells derived from human pancreatic islet beta cells. Cell Stem Cell.

[5] Cardinale V, Wang Y, Carpino G (2012). The biliary tree - a reservoir of multipotent stem cells. Nat Rev Gastroenterol Hepatol.

[6] Liu J, Liu Y, Wang H (2013). Direct differentiation of hepatic stem-like WB cells into insulin-producing cells using small molecules. Sci Rep.

[7] Yechoor V, Liu V, Espiritu C (2009). Neurogenin3 is sufficient for transdetermination of hepatic progenitor cells into neo-islets in vivo but not transdifferentiation of hepatocytes. Dev Cell.

[8] Smukler SR, Arntfield ME, Razavi R (2011). The adult mouse and human pancreas contain rare multipotent stem cells that express insulin. Cell Stem Cell.

[9] Zhou Q, Brown J, Kanarek A (2008). In vivo reprogramming of adult pancreatic exocrine cells to beta-cells. Nature.

[10] Ho JH, Tseng TC, Ma WH (2012). Multiple intravenous transplantations of mesenchymal stem cells effectively restore long-term blood glucose homeostasis by hepatic engraftment and β-cell differentiation in streptozocin-induced diabetic mice. Cell Transplant.

[11] Kim SJ, Choi YS, Ko ES (2012). Glucose-stimulated insulin secretion of various mesenchymal stem cells after insulin-producing cell differentiation. J Biosci Bioeng.

[12] Chang C, Wang X, Niu D (2009). Mesenchymal stem cells adopt beta-cell fate upon diabetic pancreatic microenvironment. Pancreas.

[13] Lin G, Wang G, Liu G (2009). Treatment of type 1 diabetes with adipose tissue-derived stem cells expressing pancreatic duodenal homeobox 1. Stem Cells Dev.

[14] Fumimoto Y, Matsuyama A, Komoda H (2009). Creation of a rich subcutaneous vascular network with implanted adipose tissue-derived stromal cells and adipose tissue enhances subcutaneous grafting of islets in diabetic mice. Tissue Eng Part C Methods.

[15] Lee J, Wen J, Park JY (2009). Reversal of diabetes in rats using GLP-1-expressing adult pancreatic duct-like precursor cells transformed from acinar to ductal cells. Stem Cells Dev.

[16] Trivedi HL, Vanikar AV, Thakker U (2008). Human adipose tissue-derived mesenchymal stem cells combined with hematopoietic stem cell transplantation synthesize insulin. Transplant Proc.

[17] Gabr MM, Sobh MM, Zakaria MM, Refaie AF, Ghoneim MA (2008). Transplantation of insulin-producing clusters derived from adult bone marrow stem cells to treat diabetes in rats. Exp Clin Transplant.

[18] Li Y, Zhang R, Qiao H (2007). Generation of insulin-producing cells from PDX-1 gene-modified human mesenchymal stem cells. J Cell Physiol.

[19] Chen LB, Jiang XB, Yang L (2004). Differentiation of rat marrow mesenchymal stem cells into pancreatic islet beta-cells. World J Gastroenterol.

[20] Yang L, Li S, Hatch H (2002). In vitro trans-differentiation of adult hepatic stem cells into pancreatic endocrine hormone-producing cells. Proc Natl Acad Sci USA.

[21] Woodbury D, Schwarz EJ, Prockop DJ, Black IB (2000). Adult rat and human bone marrow stromal cells differentiate into neurons. Neur Sci Res.

